# Mind–glucose control interplay in type 1 diabetes: management of exam-related stress using the MiniMed™ 780G insulin pump among high school students

**DOI:** 10.1186/s13098-026-02132-0

**Published:** 2026-03-18

**Authors:** Nancy Samir Elbarbary, Nahla Nagy, Lena Ahmed Ismail, Sarah Ashraf Abouelnasr, Eman Abdel Rahman Ismail

**Affiliations:** 1 Department of Pediatrics, Faculty of medicine, Ain shams University, 25 Ahmed Fuad St. Saint Fatima, Heliopolis, Cairo, 11361 Egypt; 2 Department of Psychiatry, Faculty of medicine, Ain shams University, Cairo, Egypt; 3 Faculty of Medicine, Cairo University, Cairo, Egypt; 4 Department of Clinical Pathology, Faculty of medicine, Ain shams University, Cairo, Egypt

**Keywords:** Type 1 diabetes, MiniMed™ 780G automated insulin delivery, glycemic excursions, continuous glucose monitoring, adolescents, exam stress

## Abstract

**Background:**

Academic stress can significantly impact glycemic outcome and psychological well-being in adolescents with type 1diabetes (T1D).

**Objectives:**

To evaluate the role of MiniMed™ 780G insulin pump in managing exam-induced glycemic deterioration among high school students with T1D.

**Subjects and methods:**

This prospective study included 53 adolescents with T1D using MiniMed™ 780G insulin pump. Participants were monitored during routine academic and exam periods. Psychological stress was measured using the Perceived Stress Scale (PSS-10), Hamilton Anxiety Rating Scale (HAM-A) and Hamilton Depression Rating Scale (HAM-D). Serum cortisol level was assessed and continuous glucose monitoring (CGM) data were monitored. Paired t-test and Wilcoxon test were used for parametric and non-parametric data, respectively to identify changes in study variables between study periods. A p value < 0.05 was considered statistically significant.

**Results:**

Although PSS-10, HAM-A and HAM-D scores as well as serum cortisol level (15.7 ± 4.6 versus 8.6 ± 3.1 µg/dL) were elevated in high school students with T1D during exam period compared with baseline values (*p* < 0.001); however, no significant change was found as regards mean sensor glucose (153.23 ± 14.1 versus 147.51 ± 13.7 mg/dL; *p* = 0.561), coefficient of variation (35.11 ± 8.0 versus 32.81 ± 8.2%; *p* = 0.167), time in range (TIR) (75.10 ± 8.8 versus 78.20 ± 9.0%; *p* = 0.086) and time in tight range (58.50 ± 7.0 versus 60.10 ± 7.5%; *p* = 0.311) which corresponds with the significant increase in auto basal and auto correction insulin delivery of MiniMed™ 780G (*p* < 0.001).

**Conclusion:**

The automated adaptive algorithm of the MiniMed™ 780G system mitigated exams-related stress hyperglycemia, reducing glycemic variability and successfully maintaining the recommended glycemic outcomes with a TIR greater than 70% throughout the exam period.

## Introduction

Adolescence is a critical period characterized by physiological and psychological challenges [1]. For adolescents with type 1 diabetes (T1D), these challenges are further complicated by the constant demands of diabetes self-management, particularly during periods of high emotional and cognitive stress such as academic examinations. Exam stress, a prevalent phenomenon among high school students, can significantly impact glycemic outcome through the activation of stress hormones such as cortisol and catecholamines, which increase insulin resistance and hepatic glucose output [2, 3]. This interplay of stress and glycemic instability poses a unique challenge to students with T1D who are striving to maintain optimal glucose levels while navigating high-stakes academic pressures.

Maintaining recommended glucose metrics is essential not only for short-term safety, such as avoiding hypoglycemia during testing, but also for reducing the risk of long-term complications [4]. However, traditional methods of insulin delivery such as multiple daily injections (MDI) or open loop insulin pump therapy may not provide the level of responsiveness or automation needed to handle acute fluctuations during exam period. As such, there is growing interest in the role of advanced diabetes technologies in improving outcomes for young people during such critical periods [5, 6].

The Medtronic MiniMed™ 780G system represents a new generation of automated insulin delivery (AID) systems. Unlike earlier models, the MiniMed™ 780G algorithm allows for automatic basal adjustments every five minutes and automated correction boluses to help maintain glucose levels closer to target [7, 8]. The ability of the system to respond dynamically to fluctuations in glucose, especially under unpredictable circumstances such as stress, holds promise for reducing glycemic variability and improving time in range (TIR) even in adolescents, a population known for erratic glucose profiles [9, 10].

Emerging data suggest that the use of AID systems such as the MiniMed™ 780G can lead to significant improvements in key glycemic metrics, including increased TIR and decreased time spent in hyperglycemia and hypoglycemia [11, 12]. Importantly, these benefits could contribute to improved quality of life, reduce diabetes-related distress and increase confidence in disease management among young users and their caregivers [13, 14].

Despite these advances, to our knowledge, no previous studies have examined the specific impact of AID systems on glycemic outcomes during increased academic stress such as school examinations, a time when both glucose control and cognitive performance are crucial. Stress-induced hyperglycemia can impair cognitive function, mood and test performance [15], while hypoglycemia poses immediate risks and can be a source of intense anxiety for students. Thus, ensuring reliable and stable glycemic metrics during exams is not only a medical priority but also an educational and psychological one. Therefore, this study evaluated the role of MiniMed™ 780G system in managing exam-induced glycemic deterioration among high school students with T1D and explored whether this system can help this vulnerable population maintain better glycemic outcome during exam period.

## Subjects and methods

### Study design

This prospective cohort study was designed to evaluate the impact of academic exam stress on glycemic outcome in high school students with T1D using the Medtronic MiniMed™ 780G AID insulin pump system. In our country, exam periods are distinct blocks, unlike systems where exams occur continuously. The exam period lasted one month at the end of academic year, during which students underwent formal final academic assessments. The study was divided into two distinct phases: three months baseline period representing typical school day activity (routine academic non-exam period), followed by one month exam period. The studied variables were compared within the first month of the three-month baseline period before exams to capture stable school-day routines and reduce short-term variability inherent to continuous glucose monitoring (CGM) data. Those three months were followed by a final exam month at the end of the school year which reflects concentrated stress exposure during final examinations. The two periods were comparable (one month each) and they were separated by two months to avoid the gradual increased stress as final exam period approached.

### Participants

Fifty three participants were recruited from the pediatric and adolescent diabetes outpatient clinic at Ain Shams University Hospital, Cairo, Egypt, a tertiary university hospital which serves a large population and provides specialized diabetes care. The study was approved from the local ethical committee of Ain Shams University, Faculty of Medicine (FMASU 142/2024) and all participants and/ or their legal representatives provided signed, informed consent after being informed about the study before any trial-related activities. Reporting of the study conforms to Consolidated Standards of Reporting Trials 2010 statement. The sample size was determined pragmatically based on the number of eligible adolescents attending the outpatient clinic during the recruitment period according to inclusion an exclusion criteria. Given the exploratory nature of this study and the limited availability of adolescents using AID systems during exam period, a convenience sample approach was used.

Eligible adolescents aged between 15 and 18 years and diagnosed with T1D [16] for at least one year. All participants used the Medtronic™ 780G AID system (Medtronic, Northridge, CA, USA) with Guardian™ 4 calibration-free sensor MiniMed and Guardian link transmitter initiated at least 6 months prior to study enrollment with access to the internet and a computer system that met requirements for uploading the study pump data. Insulin Aspart (NovoRapid^®^, Novo Nordisk, Copenhagen, Denmark) was used in all patients on MiniMed™ 780G AID system. Adolescents with other chronic conditions that could affect glucose metabolism or with acute or ongoing infections during the study periods were excluded. Furthermore, participants using diabetes technologies other than the MiniMed™ 780G or those using any psychiatric medication were also excluded.

### AID protocol steps

All participants had active AID features enabled, with target glucose levels set at 100 mg/dL (5.5 mmol/L)-default Medtronic ™ 780G algorithm setting- and active insulin time (AIT) set for 2 h. Insulin bolus was determined based on each participant’s insulin to carbohydrate ratio (ICR) for that given meal. Auto-correction boluses were enabled and adjusted automatically by the system algorithm as per glucose trends. Device data were collected via Medtronic CareLink™ and included CGM metrics readings, insulin delivery patterns, and system-generated automated correction boluses. Clinical and technical support was always available with text messaging and phone calls during the study period. Participants were asked to maintain their usual routines including meal patterns, physical activity and daily routines, with no changes made to pump settings unless clinically necessary.

Glucose and insulin metrics were collected and analyzed at the end of the first month of the routine academic period and at the end of exam period reflecting the whole month period. The percentages of time in range (TIR) 70–180 mg/dL (3.9–10 mmol/L), time in tight range (TITR) 70–140 mg/dL (3.9–7.8 mmol/L), time below range (TBR) 70 mg/dL (3.9 mmol/L) and 54 mg/dL (3.0 mmol/L), and time above range (TAR) 180 mg/dL (10.0 mmol/L) and 250 mg/dL (13.9 mmol/L) were calculated. CGM-captured hypoglycemia was considered as one episode when glucose fell to < 70 mg/dL (3.9 mmol/L) for at least 15 consecutive minutes. Glycemic outcome was estimated as glucose management indicator (GMI) using a minimum of 14 days of CGM data. Glucose variability was estimated by the calculation of the coefficient of variation (CV) of CGM readings. These parameters were compared between the baseline and exam periods to assess the impact of academic stress on glycemic stability. CGM trend graphs for each period were reviewed to visually assess patterns of hyperglycemia or hypoglycemia during regular school days and examination time blocks.

Participants were classified according to the standardized CGM metrics cutoffs [17] during routine academic period compared with final exam period; TIR ≥ 70%, TBR < 70 mg/dL ≥ 4, TBR < 54 mg/dL ≥ 1%, TAR > 180 mg/dL ≥ 25%, TAR > 250 mg/dL ≥ 5% and CV ≥ 36%.

### Psychological assessment

Psychological assessment was done twice; during the first month of the baseline routine academic period and during final exam period to detect any change in emotional state related to academic stress. Psychological stress was assessed to quantify subjective stress levels. Participants also reported qualitative feedback on sleep quality, study load, and mood. Participants were surveyed with a standardized exam stress scale although the primary outcome remained glycemic metrics. To evaluate psychological stress and emotional status, three validated psychometric scales were used. The Cohen Perceived Stress Scale (PSS-10), a widely used instrument for assessing perceived stress over the past month, was administered at the end of each study phase. The PSS-10 consists of 10 items rated on a 5-point Likert scale (0 = never to 4 = very often), with total scores ranging from 0 to 40. Higher scores reflect greater perceived stress [18]. The scale was administered in the participants’ native language using validated translations [19].

Additionally, emotional well-being was assessed using the Hamilton Anxiety Rating Scale (HAM-A) and the Hamilton Depression Rating Scale (HAM-D). Each administered by a trained clinical psychologist or a study physician. The HAM-A includes 14 items measuring both psychological and somatic symptoms of anxiety, with each item scored on a scale from 0 (not present) to 4 (severe), yielding a total score from 0 to 56. A score ≥ 18 indicates mild to moderate anxiety [20]. The HAM-D, consisting of 17 items, was used to assess depressive symptoms, including mood, guilt, insomnia, and somatic concerns, with scores ≥ 17 indicating moderate depression [21].

### Serum cortisol measurement

Determination of serum cortisol levels was done using Cobas e411 with electrochemiluminescense technique (Roche Diagnostics, Mannheim, Germany). Cortisol level was assessed twice; within the first week of routine academic period and the first week of final exam period. Blood samples for serum cortisol were collected between 8 and 9 am for both study periods to take into consideration circadian variation.

### Study endpoints

The primary goal was to determine whether the 780G system could sustain glycemic stability during periods of increased academic stress indicated by TIR change, measured by CGM, from baseline routine academic period to final exam period. Secondary outcomes included changes in TITR, average sensor glucose (SG) readings, GMI, total daily insulin dose (TDD) and CV together with patient-reported outcome measures (PROMs); PSS-10, HAM-A and HAM-D as well as cortisol level. Safety outcomes were the number and duration of hypoglycemic and hyperglycemic episodes assessed by TBR and TAR.

### Statistical analysis

Statistical analyses were performed using the Statistical Package for Social Science (IBM SPSS) software version 27 (Chicago, IL, USA). Data were assessed for normality using the Kolmogorov-Smirnov test. Values were presented as mean ± SD. Paired samples t-test was used for parametric data while data with non-parametric distribution were analyzed using Wilcoxon signed-rank test to identify changes in study variables between routine academic and final exam periods. For comparison of categorical variables, the chi-square test was used. Pearson correlation coefficients were used to assess the association between two quantitative variables. A p value < 0.05 was considered statistically significant.

## Results

### Participant characteristics

A total of 53 adolescents with T1D (mean age 16.5 ± 1.1 years; 53% female) completed the study. The mean diabetes duration was 6.2 ± 2.4 years, and the average duration of MiniMed™ 780G system use prior to study enrollment was 9.8 ± 1.3 months. All participants had sensor usage > 90% during the study periods (mean, 92.1 ± 7.9%), with no significant interruptions in automated insulin pump delivery indicated by low exit rate from AID system 1.0 ± 0.8 (n/week) per patient.

### Effect of academic stress on psychological outcomes and cortisol levels

**Fig. 1:**
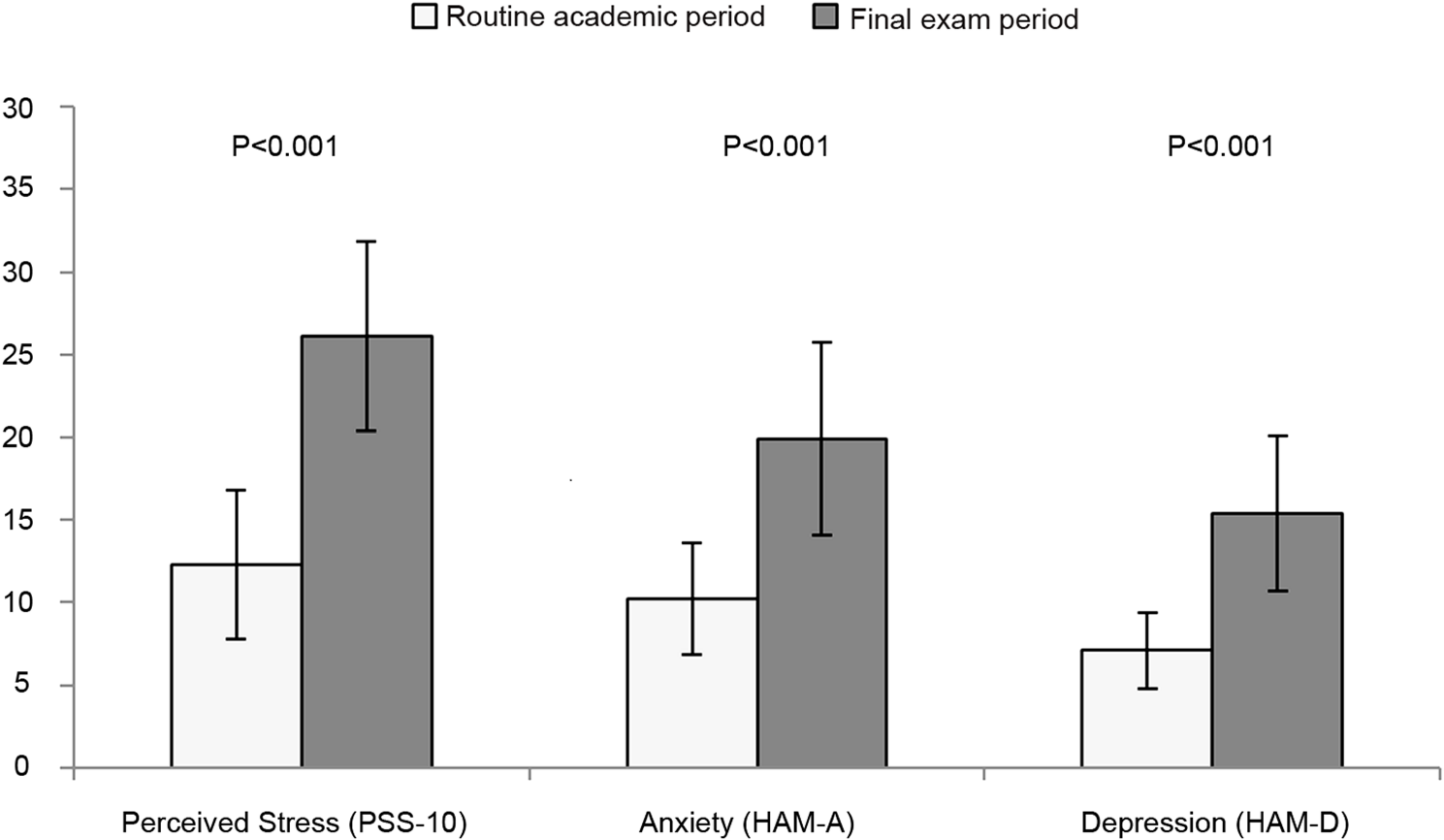
Stress level among high school students with T1D on MiniMed™ 780G insulin pump during routine academic period and final exam period

Participants with T1D reported significantly higher levels of stress and mild emotional changes during the final exam period (Fig. 1). Scores on the PSS-10 rose markedly from a baseline mean of 12.3 ± 4.5 to 26.1 ± 5.7 during exams (*p* < 0.001), reflecting elevated stress level. Similarly, the HAM-A showed a significant increase, with mean scores rising from 10.2 ± 3.4 at baseline to 19.9 ± 5.8 during exams (*p* < 0.001). Although most participants remained within the mild anxiety range, 20% scored within the moderate category. The HAM-D also showed a significant increase during exams compared with levels during routine academic period, with mean scores rising from 7.1 ± 2.3 to 15.4 ± 4.7 (*p* = 0.001); however, none of the participants met the threshold for moderate depression.

**Fig. 2:**
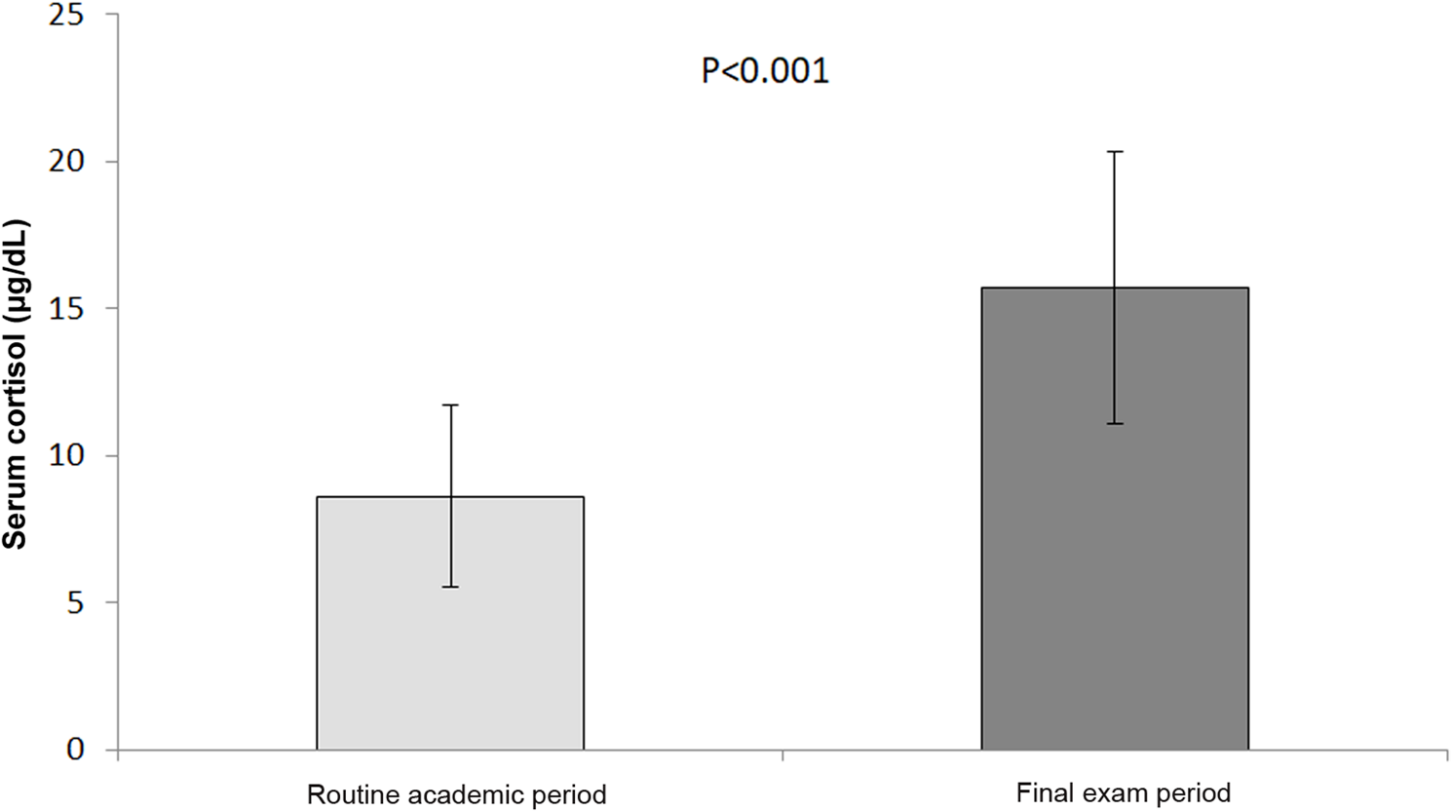
Serum cortisol level among high school students with T1D on MiniMed™ 780G insulin pump during routine academic period and final exam period

As shown in Fig. 2, serum cortisol levels were significantly elevated during exams compared with baseline levels (15.7 ± 4.6 versus 8.6 ± 3.1 µg/dL; *p* < 0.001).

### Effect of academic stress on glycemic outcomes

**Fig. 3:**
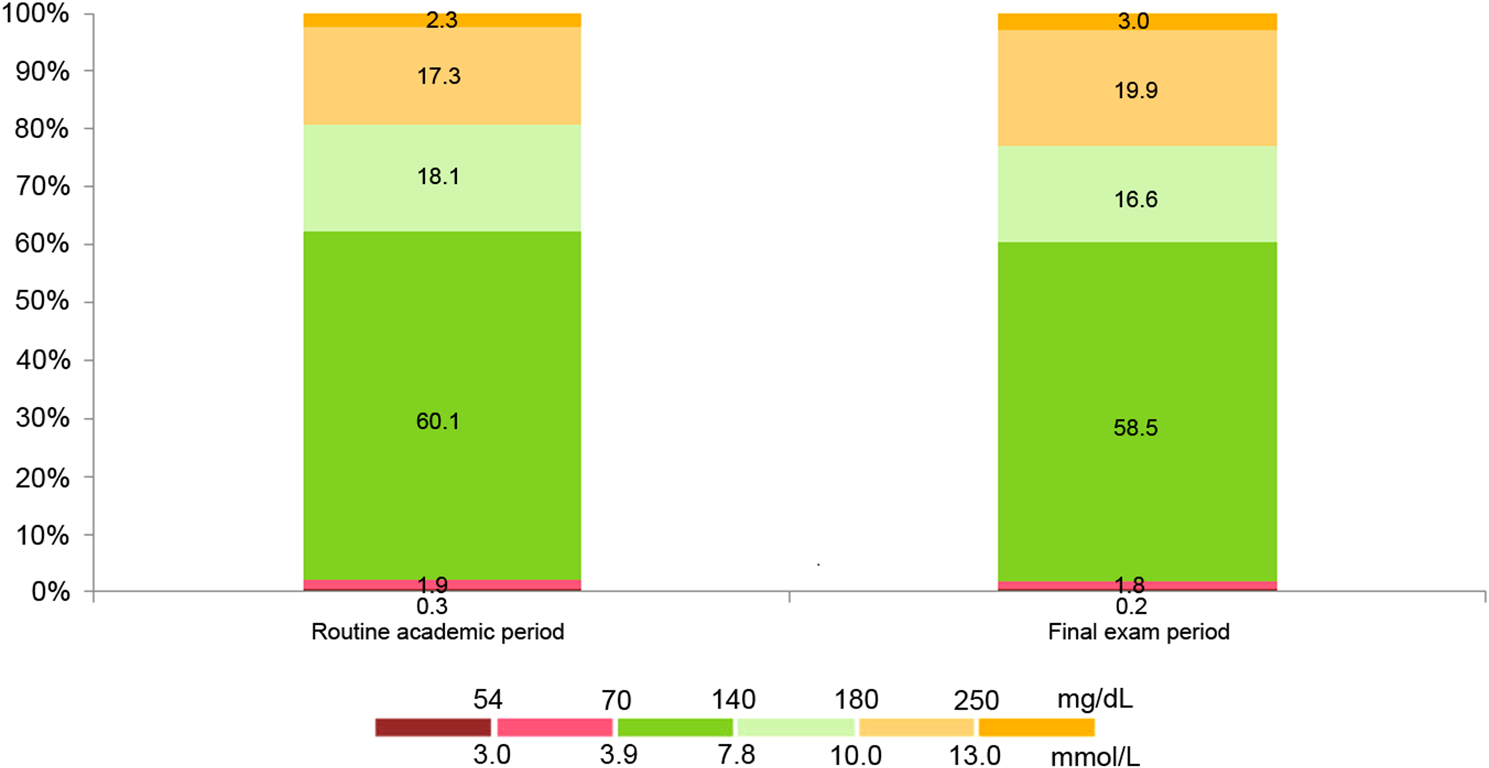
MiniMed™ 780G system performance showing the percentage of time spent in glucose ranges among high school students with T1D during routine academic period and final exam period

Comparative analysis of MiniMed™ 780G data between routine academic and final exam periods revealed no statistically significant difference as regards glucometrics and glycemic excursions (Table 1). During the final exam period, AID parameters demonstrated no statistical significance compared with baseline values; the average SG (147.0 ± 13.7 versus 153.0 ± 14.1 mg/dL; *p* = 0.561) and CV (32.8 ± 8.2 versus 35.1 ± 8.0%; *p* = 0.167) showed no statistically significant elevation during exam period while TIR (78.2 ± 9.0 versus 75.1 ± 8.8%; *p* = 0.086) and TITR (60.1 ± 7.5 versus 58.5 ± 7.0%; *p* = 0.311) showed no statistically significant decline (Fig. 3). No statistically significant changes also observed for TBR < 70 mg/dL (*p* = 0.518) or TBR < 54 mg/dL (*p* = 0.122) during the exam period compared with routine academic period. Similarly, TAR showed no significant deviation in either the 180–250 mg/dL (*p* = 0.061) or the > 250 mg/dL (*p* = 0.057) categories.

**Table 1:** Comparison between high school students with T1D on MiniMed 780G™ system as regards glucometrics and glycemic excursions during routine academic period and final exam period

Variable	Routine academic period (*n* = 53)	Final exam period (*n* = 53)	*p*-value
**Average SG (mg/dL)**	148.0 ± 13.7	153.0 ± 14.1	0.561
**GMI (eA1C %)**	6.8 ± 0.5	7.0 ± 0.5	0.912
**GMI (eA1C mmol/mol)**	50.3 ± 7.0	52.8 ± 7.1	0.081
**CV (%)**	32.8 ± 8.2	35.1 ± 8.0	0.167
**TIR 70–180 mg/dL (%)**	78.2 ± 9.0	75.1 ± 8.8	0.086
**TITR 70–140 mg/dL** (%)	60.1 ± 7.5	58.5 ± 7.0	0.311
**TBR < 70 mg/dL (%)**	1.9 ± 0.9	1.8 ± 1.0	0.518
**TBR < 54 mg/dL (%)**	0.3 ± 0.1	0.2 ± 0.1	0.122
**TAR 180–250 mg/dL (%)**	17.3 ± 7.1	19.9 ± 6.7	0.061
**TAR > 250 mg/dL (%)**	2.3 ± 1.8	3.0 ± 1.9	0.057
**Total daily dose (U/day)**	57.8 ± 11.3	68.3 ± 13.1	< 0.001
**Bolus amount (U/day)**	30.9 ± 8.4	35.8 ± 8.2	0.002
**Auto correction amount (U/day)**	13.4 ± 2.6	17.3 ± 2.7	< 0.001
**Auto Basal/Basal amount (U/day)**	26.9 ± 8.2	32.5 ± 9.1	< 0.001
**SmartGuard / Auto Mode (% per week)**	91.3 ± 20.0	92.2 ± 21.1	0.917
**Sensor wear (%)**	87.9 ± 17.8	92.0 ± 18.2	0.256
**Exit from AID per patient (n/week)**	1.0 ± 0.5	1.0 ± 0.6	0.814

T1D: type 1 diabetes; AID: automated insulin delivery System; SG: sensor glucose; GMI: glucose management indicator; eA1C: estimated A1C; CV: coefficient of variation; TIR: time in range; TITR: time in tight range; TBR: time below range; TAR: time above range

Classification of the study population according to the standardized CGM metrics cutoffs during routine academic period compared with final exam period showed that 100% of the participants maintained TIR ≥ 70% during both study periods while none exceeded TBR thresholds or TAR > 250 mg/dL threshold (*p* = 1.000). However, only 3 (5.7%) adolescents with T1D had a TAR > 180 mg/dL ≥ 25% during final exam period compared with 0% during routine academic period (*p* = 0.241). Four (7.5%) participants had a CV ≥ 36% during routine academic period compared with 7 (13.2%) during final exam period; *p* = 0.532.

The insulin delivery metrics of MiniMed™ 780G showed statistically significant changes as regards TDD, average total basal and daily automated correction boluses delivered by the 780G system. Specifically, the auto basal insulin was significantly increased from 26.9 to 32.5 U/day (*p* < 0.001), while auto correction boluses were increased from 13.4 to 17.3 U/day (*p* < 0.001). These adjustments contributed to an overall rise in TDD from 57.8 ± 11.3% to 68.3 ± 14.1 U/day (*p* < 0.001). Weight-adjusted TDD (U/kg/day) was 1.2 ± 0.31 U/Kg/day during routine academic period compared with 1.4 ± 0.36 U/Kg/Day during final exams (*p* = 0.004). The user-initiated bolus was 17.5 ± 3.0 U/day during routine academic period compared with 18.5 ± 3.5 U/day during exam period (*p* = 0.217).

### Correlation analysis

Correlation analysis during final exam period (Table 2) revealed a significant negative correlation between TIR and each of PSS-10 scores (*r* = -0.561, *p* = 0.001) and serum cortisol levels (*r* = -0.642, *p* < 0.001). Similarly, TITR was also negatively correlated to PSS-10 score (*r* = -0.576, *p* < 0.001). Moreover, glycemic variability as indicated by CV was positively correlated to PSS-10 scores (*r* = 0.711, *p* < 0.001), HAM-A scores (*r* = 0.622, *p* < 0.001) and cortisol levels (*r* = 0.539, *p* < 0.001).

**Table 2:** Correlation between stress level and the studied CGM parameters in high school students with T1D on MiniMed 780G™ system during final exam period

Variables	PSS-10 scores	
	*r*	*p* value
**Average SG** (mg/dL)	0.135	0.112
**GMI** (eA1C%)	0.117	0.815
**GMI** (eA1C mmol/moL)	0.142	0.624
**CV** (%)	0.711	< 0.001
**TIR 70–180 mg/dL** (%)	-0.561	< 0.001
**TITR 70–140 mg/dL (%)**	-0.576	< 0.001
**TBR < 70 mg/dL** (%)	0.021	0.611
**TBR < 54 mg/dL** (%)	0.076	0.781
**TAR 180–250 mg/dL** (%)	0.113	0.571
**TAR > 250 mg/dL** (%)	0.105	0.432
**Total daily insulin dose** (U/Kg)	0.156	0.241
**Basal insulin** (U/Kg)	0.068	0.312
**Bolus amount** (U/day)	0.183	0.293
**Serum cortisol** (µg/dL)	-0.642	< 0.001

SG: sensor glucose; GMI: glucose management indicator; CV: coefficient of variation; TIR: time in range; TITR: time in tight range; TBR: time below range; TAR: time above range

## Discussion

In this study, we examined the effects of academic stress on glycemic outcomes in adolescents with T1D using MiniMed™ 780G insulin pump. Our results indicated that stress levels significantly worsened during the final exam period. The mean PSS-10 score was increased, indicating a shift from moderate to high perceived stress in most participants. Similarly, anxiety, as measured by the HAM-A, increased significantly with 20% of participants meeting the criteria for moderate anxiety during exams. The HAM-D also represented a significant shift in mood, suggesting that academic stress can negatively impact psychological well-being. Furthermore, we found higher cortisol levels during exams compared with baseline levels during routine academic period. Serum cortisol level is an established biomarker of academic stress that is significantly correlated with perceived stress scores [22–24].

A longitudinal study conducted by Ahmed et al. [25] on 100 academic students aged 17–24 years found a significant increase in stress level three days before the examination, compared to five months before the examination which in turn affected both blood pressure and memory functions. Prior research emphasizes the interplay between physiological stress responses and academic outcomes, emphasizing the need for stress reducing measures to help student success [26].

In terms of psychological well-being, the significant increase in perceived stress, anxiety, and depression scores align with the understanding that stressful life events can be particularly challenging for adolescents with chronic conditions like diabetes. It has been reported that the physiological stress response is associated with both elevated glucose levels and heightened psychological distress in adolescents with diabetes [27, 28]. It is also well-documented that poor psychological health in adolescents with T1D can lead to worse clinical outcomes, including poorer glycemic outcome and increased risk of complications [29, 30].

Previous studies highlighted the influence of academic stress on diabetes management, particularly the exacerbation of hyperglycemia [31]. The parallel rise in PSS-10 and HAM-A scores in our study emphasizes stress as a dual threat; firstly on the behavioral level by disrupting sleep and causing erratic eating and secondly on the physiological level leading to catecholamine-induced insulin resistance. It has been shown that stress-related hormonal fluctuations, such as increased cortisol and adrenaline can antagonize insulin action, accelerate hepatic gluconeogenesis [32] and adversely affect glucose metrics and variability in individuals with T1D [33, 34]. This could explain why some isolated technological optimization such as open loop regimens falls short. The limitations of open-loop regimens in this context highlight a critical gap as without the automated, real-time adjustments of a closed-loop system, manual management of basal adjustments struggle to keep pace with the complex metabolic-psychological interplay triggered by academic pressure.

We found a significant correction between PSS-10 scores and TIR, TITR as well as CV. Our findings reinforce the bidirectional relationship between mental health and diabetes, especially in adolescence, a developmental stage already marked by psychosocial challenges, and emphasize the importance of a holistic approach to diabetes management, incorporating both optimization of recommended glucose metrics and psychological support during exam period.

In our study, although there was a significant elevation in perceived stress, anxiety, and depression scores as well as serum cortisol levels during exam period which could lead to decrease in TIR and increase in average SG, CV and TAR; however, these changes was mitigated by the auto correction feature in AID. The MiniMed™ 780G system’s fully automated adaptive algorithm effectively reduced glycemic variability caused by exam-related stress, maintaining recommended glycemic targets with TIR consistently above 70% without an increase in TBR during exam period. Our data demonstrated that the MiniMed™ 780G AID system could effectively minimize glycemic worsening during exams which increases our understanding of their clinical utility in stress-prone populations as adolescents with T1D. While AID algorithms are designed to autonomously counteract dysglycemia, our data revealed maintaining glucose stability during exams where stress and anxiety levels were remarkably high.

Generally, AID system improved the quality of life and sleep in people with diabetes [35, 36]. Cyranka et al. [37] showed that patients with T1D undergoing transition from MDI and self-monitoring of blood glucose (SMBG) to the MiniMed™ 780G AID system had higher levels of stress than the general healthy population but had better coping strategies and self-efficacy. The use of AID system significantly improved psychological well-being and quality of life within 3 months of transition which may be related to the significant improvement in glycemic outcomes and reduced burden of diabetes self-management. This significant improvement in quality of life was also maintained during a one-year of MiniMed™ 780G system use with improvement in sleep quality and decreased anxiety level and diabetes-related emotional distress [38, 39].

Moreover, the MiniMed™ 780G AID system has been proven to be safe and effective in improving glycemic outcomes and reducing glycemic variability among adolescents with T1D in different settings such as camp setting [40], hospital environment [41], Ramadan fasting [42] and stress-induced glucose variability during phases of menstrual cycle [43, 44].

Here in this study, we showed the performance of MiniMed™ 780G during academic stress. Our study adds to literature by using an advanced AID system, demonstrating the nuanced interaction between technology and stress in managing diabetes. In individuals with T1D, periods of stress; such as academic exams for high school students; can trigger glycemic excursions due to heightened cortisol and catecholamine responses, further complicating glucose management [31, 45, 46]. The MiniMed™ 780G system dynamically responds to such fluctuations by automatically adjusting basal insulin delivery and providing automated correction boluses every five minutes using its SmartGuard algorithm [47]. This adaptive insulin delivery can mitigate stress-related glycemic variability, potentially reducing the metabolic consequences of stress-induced inflammation.

In our study, user-initiated bolus did not differ during routine academic period compared with final exam period. Clinical trials and real-world studies of hybrid and AIDs have demonstrated that auto-correction features respond dynamically to rising glucose levels and increased glycemic variability by augmenting insulin delivery, while generally maintaining hypoglycemia within recommended targets [17]. Specifically, the MiniMed™ 780G system has been shown to deliver frequent automated correction boluses in response to sustained hyperglycemia as part of its adaptive algorithm design [8, 48]. Taken together, these findings suggest that periods of heightened psychological or physiological stress may be associated with increased automated algorithm-driven insulin delivery among users of AID systems. This pattern likely reflects adaptive algorithmic responses to stress-related glycemic excursions rather than proactive, stress-driven behavioral adjustments by users during examination periods. Overall, this points to a lack of synergistic interaction between user behavior and AID algorithm performance, with users relying predominantly on automated algorithm-driven insulin delivery during periods of markedly elevated perceived stress and cortisol levels.

Intercurrent illness represents a form of physiological stress associated with activation of counter-regulatory hormonal pathways, including increased secretion of cortisol and catecholamines which can contribute to hyperglycemia through enhanced hepatic glucose production and transient insulin resistance. While academic stress is distinct from acute illness, both conditions share common stress-related metabolic pathways that may influence glucose regulation. Notably, these findings somewhat diverge from current ISPAD guidelines [49] which recommend switching AID systems to manual mode during intercurrent illness to allow closer clinical oversight and individualized insulin adjustments. In our study, although conducted in the context of academic rather than infectious stress, glycemic metrics remained non-significant during periods of increased psychological stress among AID users. The algorithm’s stable performance, particularly under exam-related stress, indicates its capacity to maintain excellent glycemic control without the need for additional manual intervention. These findings do not contradict existing guidelines but suggest that, in selected and clinically stable situations, AID systems may be able to adapt to stress-related glycemic challenges. Further studies are required to determine whether and how these observations might inform future guideline refinements.

In this study, we aimed to contextualize our findings within the broader landscape of commercially available AID systems. In recent years, multiple hybrid closed‑loop technologies have become available, including Tandem t: slim X2 with Control‑IQ™, Omnipod 5, as well as systems developed by Ypsomed and Medtrum, each with distinct algorithmic approaches and clinical utility profiles [50, 51]. Comparative observational data indicate that glycemic outcomes, such as TIR and glucose variability, can differ between AID platforms, with some studies reporting modest advantages in metrics like TIR for the MiniMed™ 780G system compared with Tandem’s Control‑IQ algorithm over prolonged use [52]. Real‑world observational analyses have also demonstrated clinically meaningful improvements in glucometrics with the Omnipod 5 system [53].

These differences are underpinned by fundamental distinctions in algorithm design [54]. For example, the SmartGuard™ algorithm used in the MiniMed™ 780G system delivers automated basal insulin and micro‑correction boluses at frequent intervals, typically every five minutes, and allows adjustable glucose targets tailored to individual needs [55]. In contrast, other systems such as Control‑IQ employ predictive modeling with less frequent automated corrections and different programmable targets and control parameters, while systems like Omnipod 5 integrate distinct tubeless hardware designs with their own control logic and sensor integration strategies. These algorithmic and design differences can translate into variability in how each system responds to glucose excursions, behavioral inputs and user interaction patterns, which has important implications for glycemic outcomes across diverse user populations [56].

Notably, AID systems are not uniformly available across all regions or clinical settings, and in our practice environment the MiniMed™ 780G system is the most widely adopted technology. For this reason, one of the inclusion criteria for the current study was exclusive use of the MiniMed™ 780G platform. By focusing on a single AID system, we were able to minimise confounding related to inter‑platform algorithmic variability and more precisely characterize within‑individual behavioral and glycemic responses to sustained academic stress. While this approach strengthens internal consistency, it also means that extrapolation of our findings to other AID technologies should be undertaken with caution and represents an important avenue for future research.

It is worth to mention that although the observed increase in TDD during the exam period could be primarily driven by automated basal and correction insulin delivery reflecting adaptive algorithmic responses to stress-related glucose excursions; yet, alternative explanations should be considered. Changes in meal timing, dietary composition, physical activity and daily routines are common during exam periods and may have contributed to altered insulin requirements. As these factors were not objectively measured, the observed changes in TDD should be interpreted as associations rather than direct effects of academic stress alone.

### Limitations

While the current study provides valuable insights, some limitations should be considered. The sample size was relatively small and the study was conducted in a single center with reliance on a single AID technology system (Medtronic) which may limit the generalizability of the findings. Although participants were instructed to maintain their usual diet, physical activity and daily routines throughout the study; however, these factors were not objectively measured and may have contributed to changes in TDD, independent of exam stress. Future larger, multicenter prospective studies are warranted to evaluate the capacity of AID systems to adapt in response to increased insulin resistance and glycemic variability observed in the stressful exam periods and other real-world stress conditions.

## Conclusion

This prospective study highlights a critical window of a vulnerable period often overlooked in clinical diabetes management which is the academic exam stress. Optimizing outcomes demand hybrid solutions merging advanced technology with psychosocial support to address the mind-body interplay in T1D. Our data emphasize the significant impact of academic stress on both glycemic outcome and psychological well-being in adolescents with T1D, where the use of an advanced AID system like the MiniMed™ 780G was associated with maintenance of glycemic metrics, including TIR, and stable hypoglycemia risk, despite elevations in stress and cortisol levels. The MiniMed™ 780G also mitigated the stress-induced elevation in glucose variability. While the system appeared to adapt insulin delivery through auto-basal and correction boluses, these observations represent associations in an observational cohort and should be interpreted with caution. This brings attention to the need for continuous refinement in diabetes technology and the need for psychological support as an integral component of diabetes care to address challenges faced by adolescents with T1D. Schools and clinicians may consider strategies to reduce stress and support psychological well-being during high-stress periods. The use of AID systems is essential to manage blood glucose in challenging scenarios and future research with larger cohorts, comparative AID systems, and detailed behavioral and lifestyle data is warranted to better understand stress-related glucose fluctuations and optimize management in adolescents with T1D.

## Data Availability

The data that support the findings of this study are available from the corresponding author upon reasonable request.
